# Evaluation of Trichodysplasia Spinulosa Polyomavirus in Skin Biopsies, United States

**DOI:** 10.3201/eid3210.261264

**Published:** 2026-10

**Authors:** Ivan M. Espinal, Gregory W. Charville, Lauren Lawrence, Ryanne A. Brown, Kerri E. Rieger, Sebastian Fernandez-Pol

**Affiliations:** Stanford University, Stanford, California, USA (I.M. Espinal, G.W. Charville, R.A. Brown, K.E. Rieger, S. Fernandez-Pol); Guardant Health, Palo Alto, California, USA (L. Lawrence); Stanford Medicine, Stanford (R.A. Brown, K.E. Rieger)

**Keywords:** Trichodysplasia spinulosa, trichodysplasia spinulosa polyomavirus, polyomavirus, respiratory infections, viruses, inflammatory dermatoses, United States

## Abstract

Trichodysplasia spinulosa polyomavirus (TSPyV) has been identified in human endothelial cell tumors. We evaluated 25 clinical cases of various inflammatory dermatoses in California, USA, for TSPyV by using RNAscope in situ hybridization. We did not detect TSPyV in any of the biopsy samples, suggesting that endothelial TSPyV is uncommon in inflammatory dermatoses.

Trichodysplasia spinulosa polyomavirus (TSPyV) typically causes folliculocentric cutaneous eruptions, usually on the face, although the virus has been detected in tonsillar tissue, blood, urine, cerebrospinal fluid, and respiratory specimens (*1*–*4*). Recently, TSPyV was identified within the endothelial cells of tumors from 3 separate patients by analyzing off-target next-generation sequencing reads (*5*). Subsequently, in situ hybridization demonstrated the localization of TSPyV to intratumoral endothelial cells. The significance of TSPyV within endothelial cells in those cases is uncertain. Of note, all 3 patients received corticosteroids immediately before tumor resection. 

We hypothesized that TSPyV might be found in vasculitides or other cutaneous inflammatory conditions. We evaluated skin biopsy samples from 25 patients with inflammatory dermatologic conditions. Of the 25 patients, 13 received corticosteroids before skin biopsy. Among those patients, 10 were treated with corticosteroids within 10 days of the biopsy. Three patients had longer intervals between the corticosteroid dose and the biopsy, with intervals of 108 days, 1,494 days, and 1,949 days between the corticosteroid administration and the biopsy. The biopsy samples included leukocytoclastic vasculitis (n = 5), medium-vessel vasculitis (n = 1), interface dermatitis (n = 6), perniosis (n = 2), tumid lupus (n = 1), erythema nodosum (n = 4), calciphylaxis (n = 1), and nonspecific epidermal dermatoses (n =5). In addition, 1 sample of trichodysplasia spinulosa was included as a positive control.

We performed in situ hybridization by using a custom RNAScope probe (*5*) targeting the complete TSPyV genome on tumor-associated endothelial cells within 25 dermatologic samples representing a range of inflammatory conditions. We used a peptidylprolyl isomerase B RNA probe as a positive control for RNA integrity in all specimens (Figure). We did not detect TSPyV in endothelial cells in any of the 25 inflammatory skin biopsy samples. In contrast, the trichodysplasia spinulosa control had positive staining, and peptidylprolyl isomerase B staining was present in all samples (Table). 

**Figure:**
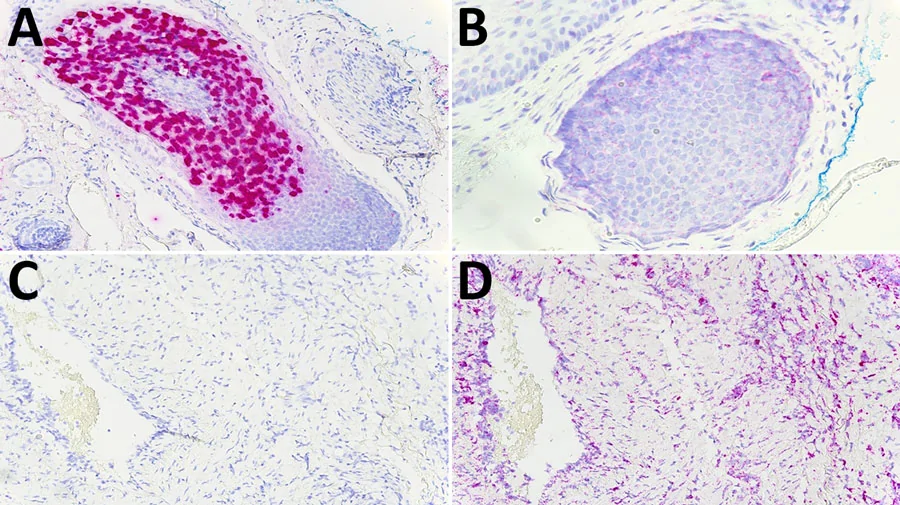
In situ hybridization (RNAscope) (*5*) for trichodysplasia spinulosa polyomavirus (TSPyV) in tissue samples, United States. A) Positive control tissue from a case of trichodysplasia spinulosa with staining for TSPyV. Original magnification ×200. B) peptidylprolyl isomerase B (PPIB) RNA probe staining on the same control tissue. Original magnification ×400. C) A representative case of inflammatory dermatosis with no staining for TSPyV RNA. Original magnification ×200. D) PPIB RNA probe staining on the same tissue in (C). Original magnification ×200.

**Table:** Cases evaluated for trichodysplasia spinulosa polyomavirus using RNAscope in situ hybridization, United States*

Case	Diagnosis	PPIB control probe result	TSPyV probe result	Days from steroid dose to biopsy
Control	Trichodysplasia spinulosa	Positive	Positive	Not applicable
1	Medium vessel vasculitis with epidermal necrosis and fibrinoid degeneration of superficial vessels (favor polyarteritis nodosa)	Positive	Negative	No steroid use
2	Erythema nodosum	Positive	Negative	No steroid use
3	Erythema nodosum	Positive	Negative	No steroid use
4	Erythema nodosum	Positive	Negative	No steroid use
5	Superficial and deep perivascular inflammation with eosinophils (favor resolving erythema nodosum)	Positive	Negative	No steroid use
6	Leukocytoclastic vasculitis	Positive	Negative	No steroid use
7	Leukocytoclastic vasculitis	Positive	Negative	No steroid use
8	Leukocytoclastic vasculitis	Positive	Negative	<1
9	Leukocytoclastic vasculitis	Positive	Negative	<1
10	Leukocytoclastic vasculitis	Positive	Negative	No steroid use
11	Interface dermatitis with eosinophils	Positive	Negative	10
12	Interface dermatitis	Positive	Negative	<1
13	Interface dermatitis with increased dermal mucin	Positive	Negative	No steroid use
14	Interface dermatitis	Positive	Negative	7
15	Interface dermatitis	Positive	Negative	<1
16	Interface dermatitis	Positive	Negative	<1
17	Perniosis	Positive	Negative	1,949
18	Perniosis	Positive	Negative	<1
19	Consistent with calciphylaxis	Positive	Negative	No steroid use
20	Epidermal ulceration with mixed inflammation and background stasis changes	Positive	Negative	No steroid use
21	Vasculopathy with focal calcium deposition (favor vasculopathy)	Positive	Negative	No steroid use
22	Ulceration with dermal necrosis, focal intravascular fibrin, and focal subtle calcium deposits (favor vasculopathy)	Positive	Negative	1
23	Neutrophilic panniculitis with fibrin thrombi and focal vessel wall calcification (favor Sweet syndrome)	Positive	Negative	1,494
24	Tumid lupus	Positive	Negative	108
25	Superficial and deep perivascular and periadnexal lymphocytic infiltrate with focal overlying vacuolar interface change and dyskeratosis, compatible with lupus	Positive	Negative	<1
*PPIB, peptidylprolyl isomerase B RNA; TSPyV, trichodysplasia spinulosa polyomavirus.				

We found no evidence of TSPyV infection in endothelial cells in this survey of cutaneous inflammatory conditions, suggesting that TSPyV does not play a role in the cutaneous inflammatory conditions we included. Of note, our cohort is small compared with that of the previous study, which initially led to the identification of TSPyV in 3 cases from 252 specimens (*3*). Therefore, the prevalence of TSPyV infection of endothelial cells might be too low to be detected in a cohort of only 25 cases. Our findings provide evidence that endothelial TSPyV infection is not commonly detected in inflammatory skin biopsy specimens and helps refine the spectrum of tissues and clinical settings in which TSPyV should be considered.
